# Recent Developments in Extracellular Matrix Remodeling for Fat Grafting

**DOI:** 10.3389/fcell.2021.767362

**Published:** 2021-12-16

**Authors:** Xin Bi, Ye Li, Ziqing Dong, Jing Zhao, Weizi Wu, Jialiang Zou, Lingling Guo, Feng Lu, Jianhua Gao

**Affiliations:** Department of Plastic and Cosmetic Surgery, Nanfang Hospital, Southern Medical University, Guangzhou, China

**Keywords:** Adipose-derived stem cells (ASCs), extracellular matrix (ECM), fat transplantation, angiogenesis, hypoxia, inflammatory cells, macrophage

## Abstract

Remodeling of the extracellular matrix (ECM), which provides structural and biochemical support for surrounding cells, is vital for adipose tissue regeneration after autologous fat grafting. Rapid and high-quality ECM remodeling can improve the retention rate after fat grafting by promoting neovascularization, regulating stem cells differentiation, and suppressing chronic inflammation. The degradation and deposition of ECM are regulated by various factors, including hypoxia, blood supply, inflammation, and stem cells. By contrast, ECM remodeling alters these regulatory factors, resulting in a dynamic relationship between them. Although researchers have attempted to identify the cellular sources of factors associated with tissue regeneration and regulation of the microenvironment, the factors and mechanisms that affect adipose tissue ECM remodeling remain incompletely understood. This review describes the process of adipose ECM remodeling after grafting and summarizes the factors that affect ECM reconstruction. Also, this review provides an overview of the clinical methods to avoid poor ECM remodeling. These findings may provide new ideas for improving the retention of adipose tissue after fat transplantation.

## 1 Introduction

Autologous fat grafting, which is widely used to augment volume and restore contour during soft-tissue reconstruction (Geeroms et al., 2019), has multiple advantages, including being minimally invasive, readily available, and inexpensive (Spear et al., 2016). Moreover, autologous fat can be easily harvested, and grafting can be performed multiple times, enabling its frequent use for filling and reshaping contour anomalies during both breast reconstruction and aesthetic operations (Ruan et al., 2019). Although the retention of grafted fat has become more predictable, it remains less than ideal (Mineda et al., 2014; Yu et al., 2015), requiring an in-depth understanding of fat regeneration after grafting and the development of various techniques to assist fat transfer (Cheriyan et al., 2014; Khouri and Khouri, 2017). The mechanism underlying the retention of transplanted adipose tissue is thought to involve a balance between successful (regeneration) and unsuccessful (cicatrization) tissue remodeling (Yoshimura et al., 2011). The morphogenesis of adipose tissue results from a self-organization process principally driven by simple mechanical interactions between adipocytes and the adipose extracellular matrix (ECM), with regeneration after injury involving the same mechanisms as regeneration after grafting (Peurichard et al., 2019). During the entire process of fat tissue regeneration, adipose ECM not only undergoes dynamic remodeling but also provides three-dimensional scaffolds for various types of cells and plays a pivotal role in optimizing outcomes, especially during the period of adipogenesis and angiogenesis (Kato et al., 2014). Therefore, improved understanding of the components, sources, and functions of adipose ECM, and optimizing the conditions affecting these factors, may facilitate the development of clinical strategies to improve the long-term retention of engrafted fat (Strong et al., 2015). This review focuses on the relationship between ECM remodeling and the microenvironment, a relatively neglected phenomenon during ECM reconstruction after fat grafting. This review also provides an overview of the potential consequence(s) of fat grafting and discusses its clinical applications.

## 2 What Happens in the Adipose Extracellular Matrix During Fat Grafting?

### 2.1 Adipose Extracellular Matrix Composition

Adipose ECM is comprised of complex structural and functional proteins, including collagen types I–VII, XVIII; non-collagenous proteins such as osteopontin, hyaluronan, and thrombospondin; and various types of adhesion proteins, such as fibronectin, laminin, proteoglycans, and elastins (Alkhouli et al., 2013; Aikio et al., 2014; Saunders et al., 2015; McKee et al., 2019).

Collagen, primarily collagen types I, III, IV, VI, and XVIII, are the most abundant proteins in the pericellular basement membrane and interstitial fibers of adipose tissue (Nakajima et al., 2002; Mariman and Wang, 2010; Mori et al., 2014; Liu et al., 2020). Type I collagen provides the main complex framework needed to maintain the structure and function of mesenchymal tissue (Liu X. et al., 2018). Type IV collagen, which is located below the vascular endothelial cell layer and acts as the basement membrane of the adipocyte region, provides binding sites for bioactive molecules, regulates cell behavior, and plays other important roles as a structural and functional protein (Streuli, 1999; Brown et al., 2011). Type VI collagen, which consists of three subunits, α1, α2, and α3, necessary for stable protein formation, provides a structural framework for adipose tissue formation (Theocharidis et al., 2016). Type VI collagen may also act as a fibrotic component that restricts adipose tissue expandability (Kokai et al., 2019). Studies in collagen VI knockout mice have shown that in the presence of expanding adipocytes, restricted ECM, mainly collagen VI, resulted in inflammation, hypoxia, and insulin resistance (Lawler et al., 2016). In addition, elimination of the increased collagen VI in adipose tissue under conditions of obesity resulted in healthier adipocytes (Sorisky et al., 2013). Type XVIII collagen is a ubiquitously expressed and structurally complex basement membrane proteoglycan, which supports pre-adipocyte differentiation and maintains the differentiation state of adipocytes (Aikio et al., 2014; Petäistö et al., 2020).

In addition to collagen, fibronectin and laminin participate in forming networks and provide attachment points for integrins anchored in the adipocyte membrane (Pope et al., 2016). Fibronectin is a ubiquitous and abundant adipose ECM protein, and a constituent of the primary mechanical structural fiber in adipose tissue (Lee et al., 2013; Zhang et al., 2020). Fibronectin has been shown to enhance cell adhesion, proliferation, and migration, as well as stem cell differentiation, including adipose conversion during adipose tissue development (Lee et al., 2013; Zhang et al., 2020). Laminin is a major component of the basement membrane, along with collagen I and collagen IV, spreading tightly over adipocytes (Vaicik et al., 2014; Zhang et al., 2020).

### 2.2 Adipose Extracellular Matrix Remodeling Process After Fat Grafting

Throughout the entire process of fat grafting, the physiological state of fat tissue is disrupted, and the ECM goes through a program involving its degradation, synthesis, and deposition. Significant changes in the ECM alter the physiological functions of the encapsulated cells, including stem cells, mature adipocytes, endothelial cells, and fibroblasts, ultimately affecting the retention rates of fat grafts (Cai et al., 2017; Zhang et al., 2020).

**FIGURE 1:**
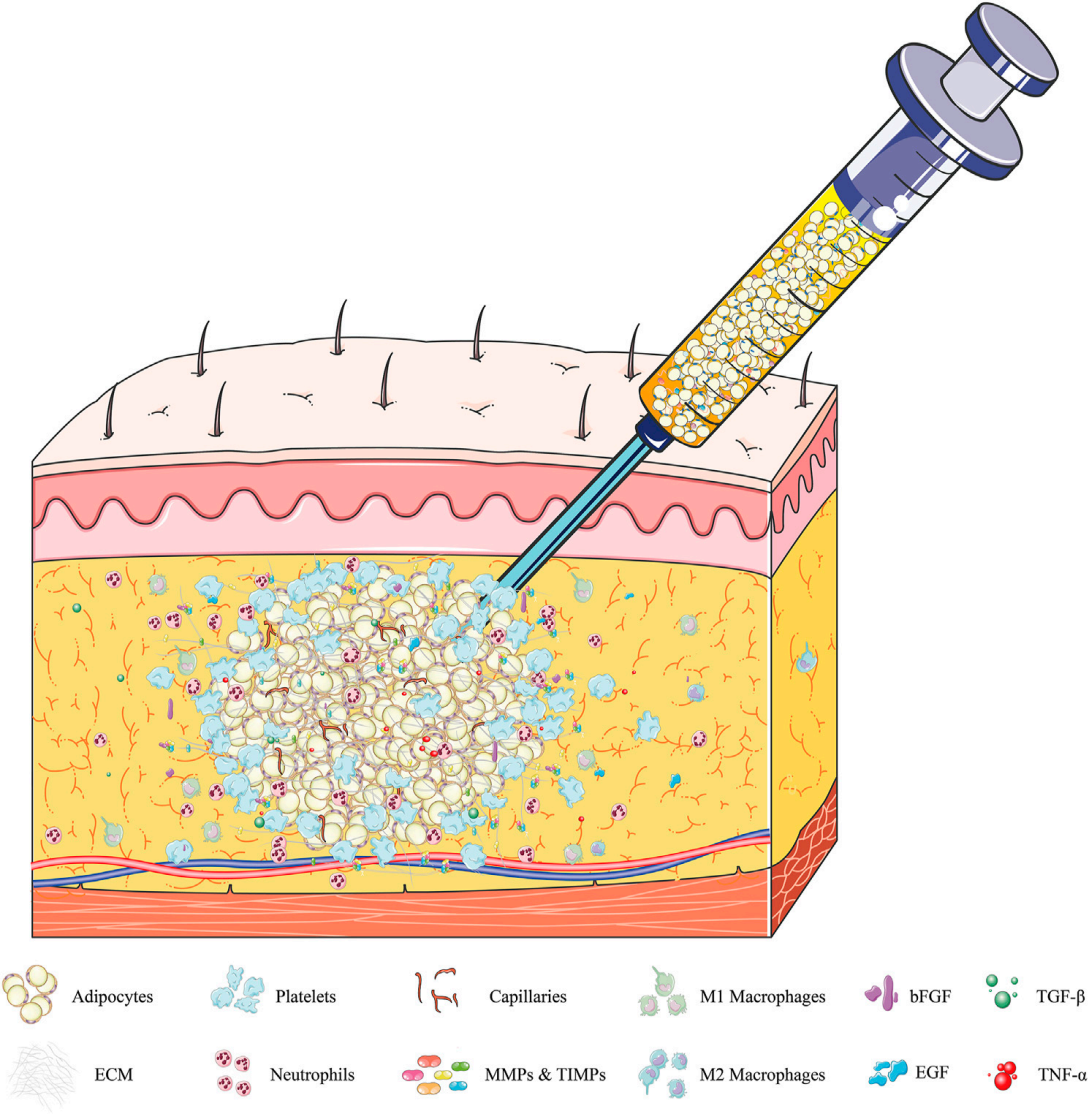
Dynamic regulation of adipose ECM within 72 h after fat grafting. Local hemorrhage after injection of fat grafts at the recipient area activates platelet cascade reactions. The adipocytes are covered by platelets and form clots within a few hours. The thin layers of fibers (fragile collagen) secreted by platelets and myofibroblasts form a loose connection between adipose lobules and “broken” ECM within 24 h. During this period, dying adipocytes, SVF, and “broken” ECM secrete various cytokines, including bFGF, TNF-α, TGF-β, and EGF; damage-related molecular pattern molecules; and some proteases, primarily MMPs and TIMPs. These factors recruit inflammatory cells (mainly neutrophils and macrophages), activate regenerative cells (such as stem cells and endothelial cells), and promote ECM synthesis, all of which play pivotal roles in the regeneration process. Abbreviations: SVF, stromal vascular fraction; bFGF, basic fibroblast growth factor; TNF-α, tumor necrosis factor-α; TGF-β, transforming growth factor-β; EGF, epidermal growth factor; MMPs, matrix metalloproteinases; TIMPs, tissue inhibitors of metalloproteinases.

**FIGURE 2:**
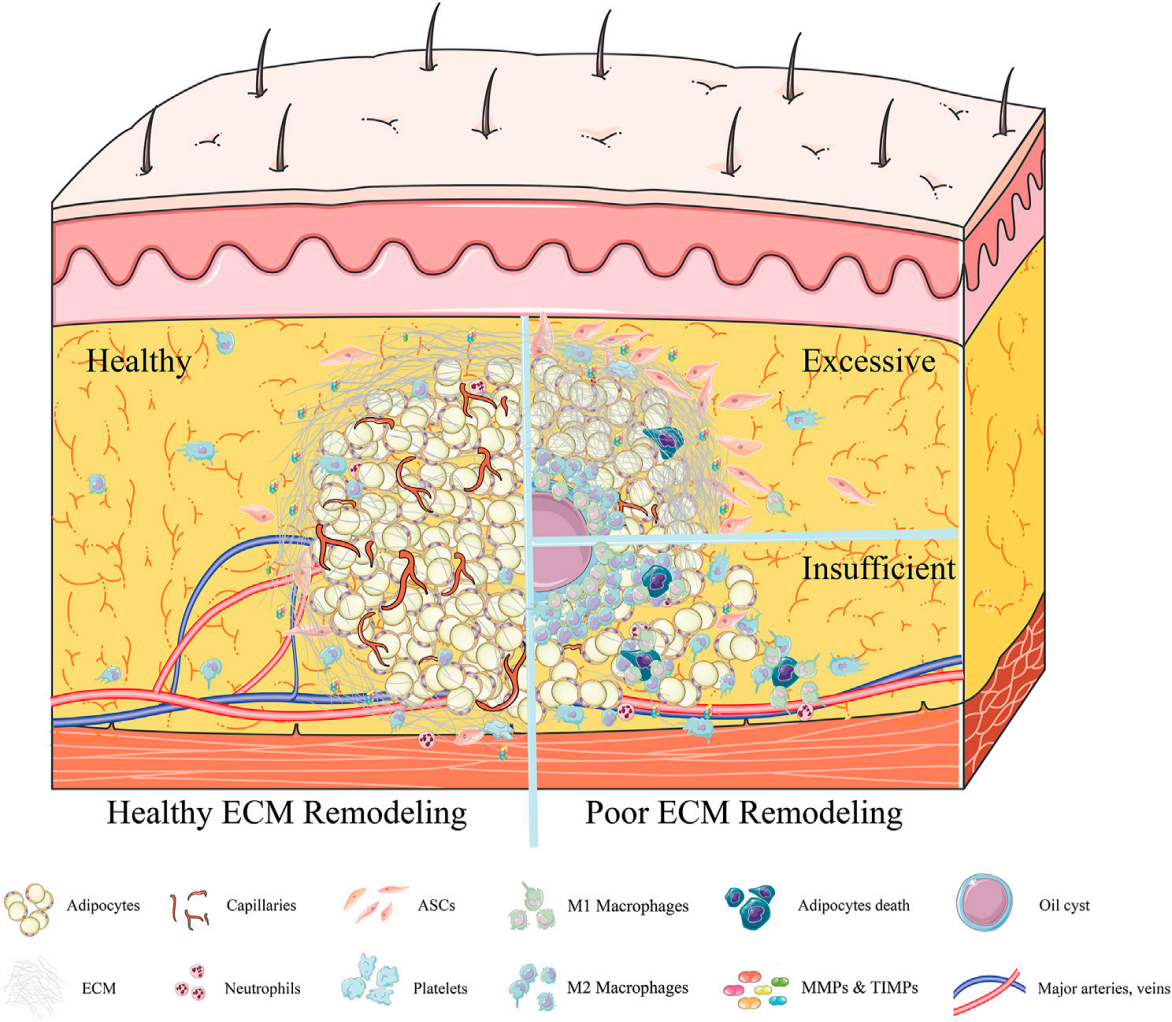
Adipose ECM remodeling ≥7 days after fat grafting. In an environment of healthy adipose ECM remodeling, the establishment of a new vascular network is accompanied by gradual thickening of the fragile collagen fibers, forming a complete ECM framework **(** ***left*** **)**. Almost all adipocytes were embedded in this newly formed cytoskeleton, after which the inflammation subsides gradually. By contrast, in an environment of poor adipose ECM remodeling **(*right*)**, excessive deposition of ECM is observed, increasing adipose tissue stiffness (*right, above*). Although high tissue stiffness can promote the migration of ASCs, it will inhibit the formation of new blood vessels. Due to poor neovascularization, oil cysts form and are surrounded by macrophages. Alternatively, the insufficient synthesis of ECM (*right, below*) can result in adipose lobules not being tightly embedded in the ECM framework. These floating fat cells will release oil droplets after necrosis, recruiting an excess number of macrophages and inducing chronic inflammation.

Specifically, liposuction at the donor site disrupts the integrity of adipose tissue, with ECM fibers cut into small pieces, and some mature adipocytes and partly regeneration-related cells detaching from the highly complex framework of the ECM (Sun et al., 2013; Khouri et al., 2014a). The lipoaspirates injected into the recipient area subsequently experience severe ischemia and hypoxia. Local hemorrhage at the injured recipient tissue activates platelet cascade reactions, resulting in the release from activated platelets of platelet-derived growth factor (PDGF), epidermal growth factor (EGF), and transforming growth factor-β (TGF-β) (Aiba-Kojima et al., 2007). The surviving adipocytes are covered by platelets, which rapidly coagulate, forming a clot at the injection site within a few hours. The thin layers of fibers (fragile collagen) secreted by platelets form a loose connection between adipose lobules and each other within 1 day (Cai et al., 2017). Simultaneously, basic fibroblast growth factor (bFGF), tumor necrosis factor-α (TNF-α), TGF-β, EGF, damage-associated molecular pattern molecules, and some proteases are likely released by dying cells, as well as by the “broken” ECM and injured host tissue (Soulez et al., 2010; Peurichard et al., 2019). These factors activate mesenchymal stem cells, including adipose-derived stem cells (ASCs) and bone marrow mesenchymal stem cells (BMSCs), which recruit inflammatory cells (mainly neutrophils and macrophages) and endothelial progenitor cells, all of which play pivotal roles in the repair process (Suga et al., 2010; Yoshimura et al., 2011; Kato et al., 2014). For example, ASCs cooperate with pericytes and endothelial cells, both cells being partly derived from ASCs, to induce neovascularization in the grafts and form larger vessels that connect the grafts and recipient areas (Han et al., 2018). Then infiltrating neutrophils, granulocytes, and macrophages promote the clearance of free oil and the phagocytosis of dead cells following fat grafting, enhancing angiogenesis and adipogenesis (Phipps et al., 2015; Liu et al., 2018a). Moreover, these inflammatory reactions significantly stimulate the synthesis of several ECM proteins, including collagen, fibronectin, laminin, and elastins, by surviving cellular components, such as ASCs, preadipocytes, and adipocytes (Mariman and Wang, 2010; Lu et al., 2011; Zhang et al., 2020). Subsequently, most fat lobules are surrounded by a temporary network of collagen fibers, which are secreted by platelets and myofibroblasts within 3 days (Luo et al., 2016) (Figure 1). Until day 7, these fragile collagen fibers gradually thicken and form a complete ECM framework, with electron microscopy showing that almost all adipocytes were embedded in this newly formed cytoskeleton in the regenerating zone (Cai et al., 2017). However, a different process is observed in the core/necrotic zone, in which adipose tissues are loosely connected and collagen fibers are more fragile, thus preventing the establishment of a complete ECM framework (Kato et al., 2014; Khouri and Khouri, 2017) (Figure 2).

During the whole remodeling process of adipose ECM, the dynamic balance between matrix metalloproteinases (MMPs) and their inhibitors, tissue inhibitors of metalloproteinases (TIMPs), plays a vital role in ECM degradation (Lin et al., 2016). MMPs are a family of calcium-dependent and zinc-containing endopeptidases that digest ECM proteins under both physiological and pathological conditions, resulting in the degradation and turnover of connective tissue and basement membrane proteins, such as collagens, proteoglycans, and elastin, as well as several circulating and cell surface components, thereby regulating cell behavior in numerous ways (Stamenkovic, 2003; Li et al., 2020). To date, 23 MMPs have been identified in human tissues; these proteins can be categorized as soluble-type (ST-MMPs) and membrane-bound type (MT-MMPs) (Sternlicht and Werb, 2001; Berg et al., 2019 Feb). Whereas ST-MMPs are secreted and diffuse directly into the ECM, MT-MMPs are bound to the plasma membrane and exert their enzymatic function(s) at the cell surface. The MMP most enriched in adipose tissue is MMP14, a key pericellular collagenase that can produce endotrophins (ETP) (Zhao et al., 2016; Li et al., 2020). ETP is a potent co-stimulator that triggers fibrosis, macrophage accumulation, and inflammation by digesting COL6α3, which accumulates in hypoxic adipose tissue (Park and Scherer, 2012). ETP can also digest collagens to prevent over-accumulation of the ECM in fat tissue and make room for new blood vessels, thus promoting the healthy regeneration of adipose tissue (Li et al., 2020). Mice with a heterozygous deletion of the gene encoding MMP-14 cannot undergo regular ECM reconstruction, reducing their fat regeneration ability (Chun et al., 2010). After fat grafting, MMP14 plays a vital role in maintaining mechanical stresses, allowing healthy ECM digestion/remodification, including ameliorated inflammation and fibrosis, as well as improved glucose and lipid metabolism (Nacu et al., 2008; Chun et al., 2010). Other MMPs are also important for degrading collagen and participate in vasculature remodeling, angiogenesis, inflammation, and atherosclerotic plaque rupture (Catalán et al., 2012). MMP-12 is the major MMP that degrades elastin in mice, whereas both MMP-3 and MMP-10 degrade fibronectin; laminin; gelatins-I, III, IV, and V; collagen fibers; and proteoglycans (Filippov et al., 2003; Ruiz-Ojeda et al., 2019). MMP-2 and MMP-9 have been shown to be necessary for degradation of adipose ECM, with rapid degradation of these damaged ECM being the first step in the angiogenic process during remodeling (Miksztowicz et al., 2017; Berg et al., 2019 Feb). Moreover, MMP-2 and MMP-9 synthesis and release are increased during adipocyte differentiation in mice. Preadipocyte treatment with MMPs-2 and -9 inhibitors markedly decreased adipocyte differentiation, suggesting that MMPs-2 and -9 could profoundly affect the behavior of cells residing on the ECM by altering ECM components (Bouloumié et al., 2001). MMP-7 and MMP-26 hydrolyze fibronectin/gelatins and digest human plasminogen, generating a fragment that inhibits angiogenesis, which may lead to poor adipogenesis after grafting (Maquoi et al., 2002; Hopps and Caimi, 2015).

MMPs are inhibited by specific endogenous TIMPs, a family of four protease inhibitors: TIMP-1, -2, -3, and -4 (Brew et al., 2000). TIMP-1 mostly inhibits ST-MMPs, whereas TIMP-2 can inhibit both ST- and MT-MMPs, with deletion of the gene encoding TIMP-2 inhibiting MMP14 (MT1-MMP)-dependent MMP2 activation (Chun et al., 2004; Kandalam et al., 2010). TIMP3 inhibits the DLK1 sheddase ADAM17 and MMP14, which has been implicated in ECM turnover, thereby modulating sDLK1 shedding and collagen I degradation (Fenech et al., 2015). TIMP3 expression is downregulated during adipogenesis and by inflammatory signals in adipocytes (Bernot et al., 2010). Although TIMPs may act as endogenous inhibitors of MMPs that are responsible for degrading excess ECM, it remains unclear whether the beneficial effects of increased TIMP activities are due solely to their suppression of MMP activities and their increase in ECM stability. Alternatively, beneficial effects of increased TIMP activities may be due to their targeting of other molecules, including ADAM (a disintegrin and metalloproteinase) and ADAMTS (ADAM with a thrombospondin type-1 motif) (Murphy, 2011).

The synthesis of ECM is the main process occurring in the recipient area during fat grafting. Relatively little is known, however, about the dominant cell type responsible for the synthesis of ECM. TGF-β1 was found to activate fibroblasts, resulting in the expression of the myofibroblast marker α-smooth muscle actin (α-SMA) and the production of ECM (Worthen et al., 2020). The fat regeneration process after grafting is partly similar to the tissue regeneration process during wound healing (Yoshimura et al., 2011; Kato et al., 2014; Horsley and Watt, 2017; Plikus et al., 2017). According to the classical view of skin wound healing, fibroblasts recruited from the dermis of intact skin adjacent to the site of inflammation promote wound healing and tissue repair by differentiating into myofibroblasts and depositing ECM (Shook et al., 2018). However, the expression of α-SMA during ECM production is not unique to a specific subset of fibroblasts. According to the fibrosis model of the liver, kidneys, and lungs, epithelial cells express α-SMA following dedifferentiation during the process of epithelial-mesenchymal transition, suggesting that epithelial cells may be the source of myofibroblasts (Iwano et al., 2002). Use of a pericyte fate mapping-technique showed that adult spinal cord scar-forming cells are derived from pericyte progeny, indicating that pericytes are the cellular origin of fibrosis (Göritz et al., 2011). Myofibroblasts expressing α-SMA may therefore differentiate from a variety of cells, including fibroblasts, astrocytes (Villesen et al., 2020), pericytes (Humphreys et al., 2010), epithelial cells (Radisky et al., 2007), endothelial cells (Soucy and Romer, 2009), and stem cells (Desai et al., 2014).

The quantitative contribution of different cell types to ECM deposition in adipose tissue is difficult to determine. Treatment of human ASCs with TGF-β1 increased their expression of α-SMA and induced their differentiation into myofibroblasts, which increased ECM gene expression (Kakudo et al., 2012), with a subset of myofibroblasts that express CD26 reported to contribute to ECM deposition (Shook and Rodeheffer, 2017). Experiments in adipose tissue obtained from obese people have shown that contact between preadipocytes and inflammatory cells in adipose tissue may contribute to the synthesis of selective ECM molecules, suggesting that preadipocytes play a role in the formation of interstitial fibrosis (Keophiphath et al., 2009). Stimulation of human preadipocytes with macrophage secretions was shown to enhance the synthesis of collagen, fibronectin, and fibrous depots (Divoux and Clement, 2011). Furthermore, cells in white adipose tissue (WAT) expressing PDGFRα, Gp38, CD29, and CD34 have strong regeneration ability, indicating that these cells have adipogenic potential and are involved in proliferation and fibrosis (Marcelin et al., 2017). Moreover, these cells can be subdivided according to their level of expression of CD9. CD9^high^ cells express genes related to ECM deposition and have a greater fibrotic ability than CD9^low^ cells (Marcelin et al., 2017). These findings indicate that the synthesis of ECM during adipose tissue regeneration is not dominated by a single cell type, but may be completed by the cooperation of multiple cell types. Among them, the myofibroblasts that express CD26 and the preadipocytes that express high levels of CD9 may be the main cell types responsible for ECM synthesis (Marcelin et al., 2017; Shook and Rodeheffer, 2017).

## 3 Factors Affecting Extracellular Matrix Remodeling

**FIGURE 3:**
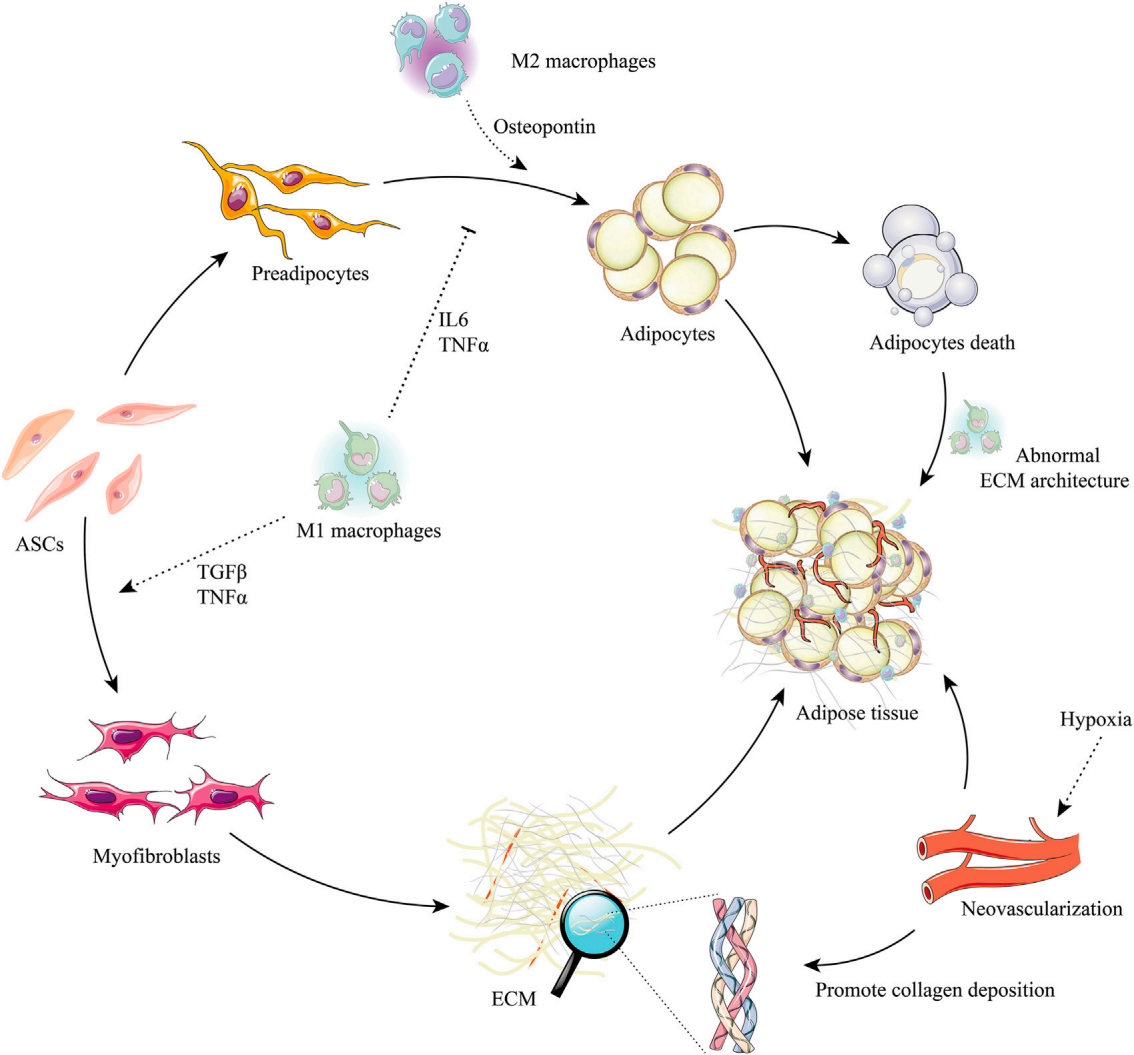
ASCs, macrophages, and the regulation of adipose ECM remodeling after grafting. ASCs have the potential to differentiate into preadipocytes and myofibroblasts. M2 macrophage signals promote the adipogenesis of preadipocytes (e.g., osteopontin) and are suppressed by M1 macrophage signals (e.g., IL-6, TNF-α). The ECM synthesis potential can trigger the myofibroblast phenotype in ASCs by M1 macrophages signals (e.g., TGF-β, TNF-α). Adipocyte death will release profuse oil droplets, which activate M1 macrophages, promote abnormal ECM deposition, and induce the formation of myofibroblasts. Hypoxia stimulates neovascularization, which is beneficial to the synthesis of ECM and promotes healthy adipogenesis. Abbreviations: IL-6, interleukin-6; TGF-β, transforming growth factor-beta; TNF-α, tumor necrosis factor-alpha; M1 macrophages, classically activated macrophage; M2 macrophages, alternatively activated macrophage.

The process of ECM remodeling after grafting is regulated by their cellular contexts and microenvironments (Hyldig et al., 2017) (Figure 3).

### 3.1 Hypoxia and Inflammation

Hypoxic conditions are critical for ECM formation and remodeling in successful soft-tissue repair (Steinbrech et al., 1999). Adipose tissue has the highest (50–60 mmHg) partial oxygen tension (pO2) among organs. After grafting, adipose tissue enters a state of severe ischemic hypoxia (such as pO_2_ < 15 mmHg) (Yoshimura et al., 2011). A recent study performed in HIF-1a knockout mice suggests that hypoxia is a powerful trigger of ECM remodeling, significantly affecting the ECM proteins synthesized by preadipocytes, as well as cell metabolism, including the secretion of high concentrations of elastase, cathepsin G, and/or MMPs (Pham, 2006; Keophiphath et al., 2009; Lu et al., 2011). Adequate hypoxia was shown to not only promote the proliferation and migration of ASCs (differentiable preadipocytes) and their secretion of growth factors (Chung et al., 2009; Chen et al., 2016; Riis et al., 2016), but to enhance the synthesis of ECM components, resulting in fibrosis (Sun et al., 2013). Hypoxia activates genes encoding collagen prolyl (P4HA1 and P4HA2) and lysyl (PLOD2) hydroxylases expression through hypoxia-inducible factor-1 (HIF-1) (Gilkes et al., 2013; Pang et al., 2020). P4HA1 and P4HA2 are necessary for collagen synthesis and regulate the proper 3D folding of newly synthesized procollagen chains (Aro et al., 2015). PLOD2 mediates remodeling of the ECM by adjusting its alignment, composition, and mechanical properties (Rosell-García et al., 2019). Hypoxia was also found to up-regulate the expression of the α-1 chain of collagen types 1, 3, and 7. The α-1 chain of collagen type 3 has been linked to the formation of type I collagen, the crucial component of the ECM (Dong et al., 2018).

Severe hypoxia may play a fundamental role in the initiation of inflammation (Crewe et al., 2017). At present, neutrophils are believed to be one of the first types of cells recruited to trauma sites and are regarded as frontier inflammatory cells that initiate the regulation of inflammation downstream of fat grafting, and to play a key role in acute inflammation after fat transplantation (de Oliveira et al., 2016; Friedman et al., 2017; Liu et al., 2018a) (Figure 1). Neutrophils react quickly with and migrate to damaged tissue sites in response to molecular chemical inducers, including (C-X-C motif) ligand (CXCL) 1 to 3, macrophage inflammatory protein-1α, C5a, and leukotriene B4 (Sadik et al., 2011; Lehman and Segal, 2020). At these sites, neutrophils phagocytize cell fragments and bacteria, forming neutrophil extracellular traps (Nets) and releasing reactive oxygen species (ROS), antimicrobial peptides, and serine proteases to remove necrotic and damaged tissue (Wilgus et al., 2013). Neutrophils also secrete large numbers of cytokines and pro-inflammatory factors, including interleukin (IL)-1β, IL-6, IL-10, monocyte chemoattractant protein-1 (MCP-1), and CXCL1, which regulate inflammatory responses and induce immune cells (Trayhurn, 2014), mainly monocytes, to migrate to the anoxic zone, where they differentiate into pro-inflammatory macrophages (Pasarica et al., 2009; O'Rourke et al., 2011; Fujisaka et al., 2013). Neutrophils were shown to regulate angiogenesis by secreting MMP-9 (Deryugina et al., 2014). Moreover, high levels of neutrophils were found to be associated with enhanced angiogenesis (Dumitru et al., 2012; Tazzyman et al., 2013; Kim and Bae, 2016), which is crucial to adipose ECM remodeling (Lee et al., 2021). However, upregulation of neutrophils was recently found to lead to increased secretion of ROS and severe tissue damage, whereas downregulation of neutrophils reduced the expression of MMP-9 and inhibited angiogenesis (Souza et al., 2013; Chakraborty et al., 2020; Fetz et al., 2021). These findings suggested that the depletion and upregulation of neutrophils during early stages of fat transplantation impaired their long-term retention, indicating that undisturbed neutrophil function is the key to initiating downstream reactions that lead to the survival and evolution of transplanted fat grafts (Liu K. et al., 2018).

Three days after fat grafting, the numbers of neutrophils began to decrease and the numbers of macrophages began to increase, with macrophages becoming the main inflammatory cell component of fat grafts (Cai et al., 2017; Cai et al., 2018; Roh and Orgill, 2018). Macrophages play essential roles in fat regeneration after transplantation by, for example, removing apoptotic and necrotic cells, regulating angiogenesis, differentiating adipocyte precursors, and, especially, regulating collagen synthesis during early stages and remodeling the ECM during later stages (Schipper et al., 2012) (Figure 2). During the process of ECM remodeling, macrophages can significantly stimulate preadipocytes to synthesize several ECM proteins (Keophiphath et al., 2009); secrete high levels of elastase, cathepsin G, and MMPs; and degrade the ECM (Lu et al., 2011). Moreover, changes in polarization and cell density can allow macrophages to secrete large amounts of type VI collagen, leading to graft fat fibrosis (Schnoor et al., 2008).

During the first week after transplantation, grafts seem to enter an “inflammatory phase”, similar to that after wound injury (Koh and DiPietro, 2011; Nissinen and Kähäri, 2015; Kim and Nair, 2019; Kloc et al., 2019). During this “inflammatory phase”, most M1 macrophages become polarized and express high levels of TNF-α, whereas pathogens and dead cells are cleared by neutrophils and macrophages (Cheng et al., 2019). M1 macrophages have phagocytic activity, produce pro-inflammatory mediators, and promote the fibrotic environment of local tissue (Werner and Grose, 2003; Wang et al., 2018). After grafting, M1 macrophages promote ECM remodeling by engulfing cellular debris, digesting damaged ECM components, and inducing the transformation of fibroblasts into ECM-secreting myofibroblasts by producing cytokines, including PDGF, TGF-β, and insulin-like growth factor (IGF)-1 in the grafts (Duffield et al., 2013). In addition, the M1 phenotype also can directly activate M1 factors, which drive a “feed-forward” state of inflammation and remodeling (IL-1b), and increase the production of MMPs and TIMPs, which drive ECM remodeling (Brown et al., 2012) (Figure 3).

Grafts are regarded as entering the “regeneration phase” 2–12 weeks after transplantation (Phipps et al., 2015). M2 macrophages gradually become the main macrophage group, producing high levels of TGF-β and secreting a large number of angiogenic factors, such as vascular endothelial growth factor (VEGF), bFGF, and MMPs, a process involving RhoA/Rho kinase signaling and the induction of endothelial cell migration (Hoang et al., 2004) (Figure 3). This period is characterized by active adipogenesis and the gradual maturation of new microvascular networks (Seaman et al., 2016; Cai et al., 2018; Roh and Orgill, 2018). In addition, M2 macrophages can regulate or reverse the abnormal accumulation of ECM by secreting MMPs and other M2 factors, such as IL-10, RELMα, and Arg-1 (Brown et al., 2012). Mounting research suggests that the ECM remodeling of transferred fat is position-dependent and that M2 macrophages dominate in the deposition of ECM (Liao et al., 2011; Cai et al., 2017; Motz et al., 2021). The SEM results suggested that single dead adipocytes in the surviving zone are phagocytized by M1 macrophages, without excessive ECM deposition (fibrosis). By contrast, a mass of dead adipocytes in the regenerating and/or necrotizing zones are surrounded by an innermost single layer of M1 macrophages and outer multilayered M2 macrophages. This process is accompanied by the clearance of free oil and the phagocytosis of dead cells by M1 macrophages, along with excessive fibrosis by M2 macrophages (Kato et al., 2014).

Depletion of macrophages from fat grafts was shown to block angiogenesis and delay ECM remodeling by reducing MMP-2 expression (Debels et al., 2013; Cai et al., 2018). Moreover, low macrophage levels resulted in the downregulation of collagen synthesis and decreased collagen types I and VI expression, thereby inhibiting ECM deposition (Cai et al., 2017). By contrast, high macrophage levels at the early stage have been associated with increased angiogenesis and hematopoietic stem cell recruitment, enhancing ECM reconstruction and improving the survival rate of fat grafts (Roh and Orgill, 2018). In addition, upregulation of macrophage levels is time-dependent, and the excessive accumulation of early inflammatory cells may lead to extreme degradation of ECM (Cai et al., 2017). In comparison, the persistence of hyper-macrophages after the “regeneration phase” of transplanted fat seems to lead to excessive fibrosis, suggesting the need to control macrophage levels within a moderate range (Mineda et al., 2014).

### 3.2 Angiogenesis

Scanning electron microscopy (SEM) has shown that ECM remodeling is strongly linked to location and blood supply (Cai et al., 2017). After grafting, the fat lobules are closely embedded in the ECM framework around the capsule, where the survival area is located, with immunostaining showing high levels of type I collagen expression around the capsule. In the regenerated area, the adipose tissue seems to be less condensed, whereas in the necrotic area, the complete ECM framework has not yet been established, and the area positive for type I collagen is significantly reduced (Cai et al., 2017). These findings suggest that ECM remodeling after fat grafting is in agreement with the classical “three-zone” hypothesis, suggesting the importance of angiogenesis to ECM deposition (Eto et al., 2012). Stimulation of hypoxia promotes neovascularization through the angiogenesis induced by hypoxia-inducible factors-1α (HIF-1α) and 2α (HIF-2α) (Zhang et al., 2010; García-Martín et al., 2016) (Figure 3). An important determinant of their difference in activity is the abundance of specific PHD enzymes, which differ in specific proline hydroxylation sites on HIFα isoforms (Appelhoff et al., 2004). Moreover, they can interact with each other to induce angiogenesis. Interestingly, HIF-1α alone is unable to induce an effective pro-angiogenic response in adipose tissue. Although HIF-1α and HIF-2α have overlapping effects on aspects of angiogenesis and ECM remodeling, increasing evidence indicates that HIF-1α and HIF-2α play unique roles in tissue regeneration (Skuli et al., 2009). HIF-1α can increase expression of VEGF, PDGF, and nitric oxide synthase (NOS) (Feng et al., 2016), whereas HIF-2α is thought to regulate the expression of Tie2 (the receptor tyrosine kinase for angiopoietin, Ang1, and Ang2), integrin, VEGF, VEGF receptor-1 (Flt-1), and VEGF receptor-2 (Flk-1), and can reduce inflammatory states (Skuli et al., 2009; Sun et al., 2013; Lee et al., 2014). Together with HIF-2α, HIF-1α facilitates cellular adaptation to hypoxia and oxygen delivery by stimulating angiogenesis, erythropoiesis, and anaerobic glucose metabolism, thereby effectively promoting adipose tissue regeneration (Rankin et al., 2007).

Neovascularization during the regeneration stage after fat grafting depends on physical ECM guidance cues and is regulated by pro-angiogenic factors, such as VEGF, PDGFs, and bFGF (Siegel-Axel et al., 2014). Specifically, ASCs secrete MMPs (mainly MMP-2 and MMP-9) and cathepsins to further break down the ECM, liberate pericytes and endothelial cells from cell niches, and convert the characteristics of the basement membrane into a pro-angiogenic environment (van Dongen et al., 2019). In addition, the degradation of ECM also releases TGF-β and various growth factors, such as VEGF and bFGF, from immobilized matrix stores (Plaks et al., 2015). As a vital regulator of physiological angiogenesis, VEGF promotes the growth of endothelial cells *in vitro* and causes angiogenesis *in vivo*. VEGF plays a key role in preventing endothelial cell apoptosis, regulating vascular permeability and inducing endothelial fenestration in some vascular beds (Lin et al., 2017). TGF-β and PDGF are important regulators in the process of maintaining proper vascular function by coverage of mural cells and facilitating vessel maturation. They can up-regulate VEGF-A mRNA expression, and might act in concert to regulate angiogenesis and ECM production (Itoh et al., 2012). In addition, PDGF can stabilize endothelial cell channels by recruiting PDGF receptor-β pericytes (Lin et al., 2017). Thus, the intimate homeostatic interactions among MMPs, pro-angiogenic factors, and other components of the ECM are critical in promoting the survival, migration, and proliferation of endothelial cells and their eventual differentiation into functional tubular networks (Van der Donckt et al., 2015).

In addition to increasing the rigidity of the vascular system, adipose ECM can directly promote or inhibit angiogenesis. Neovascularization and capillary morphogenesis after fat grafting partly depend on the balance between degradation and synthesis of the surrounding ECM (Lee et al., 2021). Formation of a stiffness-appropriate (∼2–4 kpa) network of collagen fibers within 7 days after fat grafting might promote the migration, proliferation, and differentiation of endothelial cells into functional tubular networks and the formation of functional blood vessels, which are essential for subsequent adipogenesis and tissue remodeling (Kniazeva and Putnam, 2009; Li et al., 2019). By contrast, excessive ECM deposition can affect the angiogenic properties of adipose tissue (Mongiat et al., 2016). For example, type IV collagen, a major vascular basement membrane protein, was found to inhibit initial neovascular sprouting during angiogenesis (Delaney et al., 2006). Another ECM component that plays a role in this process is type VI collagen, the main component of oil cyst walls that form after grafting. These ECM components are expected to stiffen tissue, making them less suitable for the proliferation of capillaries and the expansion of adipocytes (Kniazeva and Putnam, 2009; Kobayashi et al., 2020). Ultimately, a decrease of capillary density in new adipose tissue will negatively affect remodeling of the ECM, forming a vicious circle (Sun et al., 2013).

### 3.3 Adipose-Derived Stem Cells

ASCs are found within the stem cell niche and are surrounded by the ECM (Pope et al., 2016). In this specialized microenvironment, ECM proteins and various soluble factors regulate cell phenotype via the assembly of integrins, focal adhesions, and cytoskeletal reorganization to control the fates of ASCs (Rozario and DeSimone, 2010; Mecham, 2012; van Dongen et al., 2019) (Figure 3). ASCs, in turn, are closely associated with the composition, stiffness, and ligand pattern of the ECM (Flaim et al., 2008; Rozario and DeSimone, 2010; Choi et al., 2012; Harvestine et al., 2018).

The stiffness of adipose ECM plays a crucial role in cellular behavior, including the migration, proliferation, and differentiation of ASCs through mechanical transduction pathways. ECM deposition could increase adipose tissue stiffness, which promotes the migration of ASCs by up-regulating the expression of cell migration-related proteins CDC42, RhoA, and dynamin (Zhang et al., 2020). However, excessive stiffness could affect the differentiation of ASCs, inhibiting their ability to differentiate into adipocytes and promoting the fibrosis of adipose tissue (Li et al., 2019).

The composition of ECM proteins secreted by ASCs undergoing remodeling during adipogenesis *in vitro* changes dynamically from fibronectin-rich to laminin-rich (Zhang et al., 2019). Similar results were observed *in vivo*, in that the cells moved from the growth phase to the differentiation phase, while the ECM transitions from a fibrillar to a laminar structure (Cai et al., 2016). Laminin has been shown to enhance adipogenesis (Hoshiba et al., 2010). Upregulation of laminin expression during the early stage has been found to initially increase the expression of type I collagen, but later to decrease its expression significantly (Mariman and Wang, 2010), followed by the alteration of ECM components from fibrillary collagen types I, III, and V to basement membrane types IV and VI(Mor-Yossef Moldovan et al., 2019). The components and proteins secreted by ASCs and preadipocytes optimized ECM remodeling and produced critical biochemical and physical signals (Morissette Martin et al., 2018). Exosomes derived from human adipose mesenchymal stem cells (hASCs-Exos) can be recruited to soft-tissue wound areas of a mouse skin incision model, increasing the production of collagen types I and III during early stages (Hu et al., 2016). During later stages, exosomes may reduce fibrosis by inhibiting the expression of collagen. ASCs have the potential to differentiate into fibroblasts (Hong et al., 2013; Ebnerasuly et al., 2017; Zhou et al., 2018), with VEGF promoting the differentiation of ASCs into fibroblasts and keratinocytes *in vivo* (Zhu et al., 2021). Moreover, the addition of fibroblast growth factor (FGF)-2 and ascorbic acid-2-phosphate can permanently induce the transition of ASCs into fibroblasts *in vitro* (Adams et al., 2012). When compared with primary fibroblasts, fibroblasts differentiated from ASCs were found to produce higher levels of healthy ECM markers, including elastin, fibronectin, and type I collagen (Gersch et al., 2020). ASC supplementation in an animal model of cell-assisted lipo-transfer was found to alter inflammatory processes, promote a favorable microenvironment for angiogenesis, contribute to a more rapid recovery from hypoxia and ischemia, and reduce the excessive deposition of collagen (Hong et al., 2018).

## 4 Two Situations of Poor Remodeling of Adipose Extracellular Matrix After Grafting: Insufficient and Excessive

Dysfunction of ECM remodeling during fat grafting may significantly affect graft retention rates. Insufficient synthesis of ECM will likely reduce graft retention rates, whereas excessive deposition of the ECM does not seem to be ideal for new fat regeneration (Cai et al., 2017). Remarkably loose, disorganized adipose tissue and an increase in adipocyte size have been observed in ColVI−/−ob/ob mice, suggesting that deficient synthesis of Col VI may lead to uncontrolled adipocyte expansion and a disordered structure after grafting (Divoux and Clement, 2011). Type I collagen played a positive role in the activation of YAP, promoting the differentiation of preadipocytes into myofibroblasts, which is regarded as a key culprit in fibrosis. These findings suggested that excessive Col I may inhibit adipogenesis via activation of YAP signaling (Liu X. et al., 2018; Xu et al., 2019; Liu et al., 2020). Rather, excessive ECM deposition will likely lead to fibrosis, eventually resulting in oil cyst formation and progressive calcification, outcomes much worse than a lack of retention (Juhl et al., 2018; Ørholt et al., 2020). These situations have been reported frequently and always occur in some parts of the “regenerating zone” and throughout the “necrotic zone” (Kato et al., 2014). Predictably, oil cysts and calcifications (central fat necrosis) occur when fat is grafted in large droplets (>3 mm) and when the microenvironment around the transplanted fat does not properly improve within the first 72 h (Suga et al., 2010; Yoshimura et al., 2011). Interestingly, single necrotic adipocytes are surrounded by fat cell-sized oil droplets and are phagocytized by a single layer of M1 macrophages, leaving almost no trace. By contrast, large areas of dead adipocytes surrounded by large oil droplets are, in turn, surrounded by multiple, stratified macrophages, consisting of an internal monolayer of M1 macrophages and an external multilayer of M2 macrophages, which form a crown-like structure (Eto et al., 2012). Locally persistent inflammation results in high levels of M2 macrophages surrounding oil cysts, inducing monocytes to produce angiogenic and fibrotic cytokines, including IL-4, IL-10, IL-13, and TGF-β1 (Wick et al., 2013). Recent studies suggest that M2 macrophages may be key contributors to fibrogenesis and calcification (Braga et al., 2012; Bility et al., 2016; Hou et al., 2018). Thus, excessive ECM deposition, including fibrotic oil cysts and calcifications after fat grafting, may result from the overexpression of collagen types IV and VI, induced by upregulated M2 macrophages and fibrotic cytokines (Kim et al., 2015). The microenvironment of the walls of the oil cysts, such as the numbers of ASCs/progenitor cells and/or oxygen tension, may be insufficient for normal adipogenesis, resulting, even a few years later, in chronic inflammation in cyst walls (Mineda et al., 2014). Long-term follow-up of patients who have undergone autologous fat augmentation mammoplasty has shown that the oil cysts formed after grafting will remain problematic without surgical intervention (Tassinari et al., 2016; Juhl et al., 2018) (Figure 2).

## 5 Clinical Avoidance of Poor Extracellular Matrix Remodeling After Grafting

Effective methods are necessary to improve the early-stage microenvironment of the graft, maintain the proper ECM remodeling, and avoid complications caused by incorrect techniques. Experimental results and previous experience suggest the feasibility of several methods (Table 1).

**TABLE 1:** Clinical avoidance of poor ECM remodeling after grafting.

Method	Principle	References
Up-regulate the level of inflammation in the recipient area or supplement the transplanted recipient area with an adequate number of macrophages in the early stage	Macrophage mediated inflammation enhances the ECM protein synthesis in the early-stage	Wang et al. (2020); Phipps et al. (2015)
Down-regulate the level of inflammation in the recipient area or deplete the macrophages in the transferred fat in the later stage	Depletion of macrophages relieved the fibrosis in the transferred fat during the late-stage	Keophiphath et al. (2009); Kumar et al. (2018); Schnoor et al. (2008); Spencer et al. (2010); Anghelina et al. (2006); Wang et al. (2020); Cai et al. (2017)
Injections should not be performed without cannula movement, and the injection speed should not exceed 0.1ml/1 cm of cannula movement	Ideal revascularization depends on the graft-to-recipient interface not exceeding a maximum of 1.6 mm	Khouri and Khouri, (2017); Eto et al. (2012); Pu, (2012); Abu-Ghname et al. (2019); Khouri et al. (2014b); Khouri et al. (2014a)
Adipose-derived stem cells ASCs - assisted lipotransfer	ASCs - assisted lipotransfer can promote vascular ingrowth and accelerates revascularization between donor and recipient tissues	Yoshimura et al. (2008); Yoshimura et al. (2010); Kølle et al. (2013); Hong et al. (2018); Yoshimura et al. (2020); Marks et al. (2017)
The fat fraction in the lower liposuction high-quality fat is recommended as a suitable alternative for implantation	High-quality fat contains more ECM, ASCs, and higher anti-inflammatory and angiogenesis fat factors	Allen et al. (2013); Qiu et al. (2016); De Francesco et al. (2017); Guan et al. (2020)
Apply External volume expansion EVE on the recipient area pre-operation and post-operation	EVE can enhance tissue vascularity, induce matrix deposition, increase adipose ECM’s stiffness, regulate ASC proliferation and differentiation via shifting ECM synthesis from fibronectin to laminin	Heit et al. (2012); Lancerotto et al. (2013); Chin et al. (2016); Khouri et al. (2000); Zhang et al. (2020)

5.1 During the early stage after autologous fat grafting, circulating inflammatory cells, especially macrophages, will preferentially infiltrate the donor site. In this stage, the level of inflammation will be lower at the recipient than at the donor site, resulting in delayed repair of the recipient area, which may hinder the reconstruction of the ECM during the early stage (Wang et al., 2020). Thus, early supplementation of the recipient area with an adequate number of macrophages may increase the graft retention rate by improving angiogenesis and ECM remodeling (Phipps et al., 2015).

5.2 Chronic inflammation mediated by macrophages leads to dysfunction in ECM remodeling (Keophiphath et al., 2009; Kumar et al., 2018). Excessive macrophage infiltration can also result in the excess secretion of type VI collagen and result in fibrosis (Schnoor et al., 2008; Spencer et al., 2010). The rapid and massive infiltration and rapid evacuation of macrophages after fat grafting may lead to better tissue vascularization and facilitate ECM remodeling (Anghelina et al., 2006; Wang et al., 2020). Therefore, trying to deplete the macrophages from transferred fat during the late stage may reduce the excessive deposition of ECM and reduce fibrosis (Cai et al., 2017).

5.3 Ideal revascularization depends on the graft-to-recipient interface not exceeding a maximum of 1.6 mm (Khouri and Khouri, 2017). This interface may be optimized by a 3D distribution of the graft to reduce the obstacle of inadequate blood supply for remodeling of the ECM (Eto et al., 2012; Pu, 2012; Abu-Ghname et al., 2019). During fat injection, the cannula is first driven forward to create a tunnel in the recipient tissues, followed by fat injection during retraction, filling the tunnels with fat ribbons (Khouri et al., 2014b). Injection without cannula movement will lead to a fat blob, exceeding the optimal graft-to-recipient interface distance (Khouri et al., 2014a). Therefore, injections should not be performed without cannula movement, and the injection speed should not exceed 0.1 ml/1 cm of cannula movement (Khouri and Khouri, 2017).

5.4 Autologous fat grafting supplemented with ASCs promotes vascular ingrowth at the recipient site and accelerates revascularization between donor and recipient tissues (Yoshimura et al., 2008; Yoshimura et al., 2010; Kølle et al., 2013; Hong et al., 2018; Yoshimura et al., 2020). ASC-assisted lipotransfer provides a better blood supply for remodeling of the ECM, but large, randomized, controlled clinical trials have not yet been performed (Marks et al., 2017).

5.5 A graded density of fat can be obtained by centrifuging the products of liposuction using standard Coleman technology (Allen et al., 2013). Compared with the fat in the upper liposuction, the fat in the lower liposuction contains more ECM and ASCs, and higher concentrations of anti-inflammatory and fat angiogenesis factors (Qiu et al., 2016; De Francesco et al., 2017). The fat fraction in the lower liposuction, which is called high-quality fat, is promising for improving vascularization and ECM remodeling, and is recommended as a suitable alternative for implantation (Guan et al., 2020).

5.6 External volume expansion (EVE) can enhance tissue vascularity, induce matrix deposition, and increase the stiffness of adipose ECM, which recruits circulating mesenchymal stromal cells (Heit et al., 2012; Lancerotto et al., 2013; Chin et al., 2016). In addition, EVE can regulate ASC proliferation and differentiation via shifting ECM synthesis from fibronectin to laminin, thereby priming the recipient site for autologous fat transfer (Khouri et al., 2000; Zhang et al., 2020).

5.7 Oil cysts filled with necrotic material result in persistent inflammation, and calcification continues to develop over time, with these progressive changes persisting (Mineda et al., 2014; Shida et al., 2017). Therefore, larger oil sacs should be suctioned under ultrasound guidance, taking care to ensure that all their contents are extracted (Shida et al., 2017). If the suction is incomplete, the lump will persist and the patient will be dissatisfied. Oil cysts that are not treated effectively during the early stages will develop into calcified masses surrounded by extensive calcified capsules (Tassinari et al., 2016). These lesions can only be treated by complete lumpectomy (Ørholt et al., 2020).

## 6 Conclusion

This review summarized the sources of ECM remodeling after fat grafting, as well as the mechanisms by which the ECM interacts with surrounding cells and microenvironments during the process of fat regeneration. These results provide a greater understanding of the application of fat repair and regeneration after grafting. ECM remodeling is regulated by extracellular molecular synthesis and degradation, accompanied by physiological reactions such as tissue development and repair, as well as by pathological processes such as hypoxia and inflammation (Clause and Barker, 2013). ECM remodeling also has a profound impact on the migration, proliferation, and differentiation of surrounding cells. Additional studies are needed to further assess the composition, function, and mechanism of regeneration of adipose tissue ECM (Mouw et al., 2014). These studies may reveal the directional control of the microenvironment and the cellular conditions beneficial to ECM remodeling, providing new concepts for improving the retention rate of adipose tissue after fat transplantation. Greater efforts are needed to apply these methods clinically.
